# Chronic oxidative stress upregulates Drusen-related protein expression in adult human RPE stem cell-derived RPE cells: A novel culture model for dry AMD

**DOI:** 10.18632/aging.100516

**Published:** 2012-12-20

**Authors:** David M. Rabin, Richard L. Rabin, Timothy A. Blenkinsop, Sally Temple, Jeffrey H. Stern

**Affiliations:** ^1^ Center for Neuropharmacology and Neuroscience, Albany Medical College, Albany NY 12208, USA; ^2^ Neural Stem Cell Institute, One Discovery Drive, Rensselaer, NY 12144, USA

**Keywords:** RPE, oxidative stress, AMD

## Abstract

**Purpose:**

The goal of this study was to examine changes in the expression of transcripts and proteins associated with drusen in Age-related Macular Degeneration (AMD) after exposing human retinal pigment epithelium (hRPE) cells to chronic oxidative stress.

**Methods:**

Primary adult human RPE cells were isolated from cadaveric donor eyes. The subpopulation of RPE stem cells (RPESCs) was activated, expanded, and then differentiated into RPE progeny. Confluent cultures of RPESC-derived hRPE and ARPE-19 cells were exposed to a regimen of tert-butylhydroperoxide (TBHP) for 1-5 days. After treatment, gene expression was measured by quantitative PCR (qPCR), protein expression was assessed by immunocytochemistry and transepithelial resistance and cell toxicity were measured.

**Results:**

hRPE cells exposed to a regimen of TBHP for 5 days upregulate expression of several molecules identified in drusen, including molecular chaperones and pro-angiogenic factors. 5-day TBHP treatment was significantly more effective than 1-day treatment at eliciting these effects. The extent of hRPE response to 5-day treatment varied significantly between individual donors, nevertheless, 6 transcripts were reliably significantly upregulated. ARPE-19 cells treated with the same 5-day stress regime did not show the same pattern of response and did not upregulate this group of transcripts.

**Conclusions:**

RPESC-derived hRPE cells change significantly when exposed to repeated oxidative stress conditions, upregulating expression of several drusen-related proteins and transcripts. This is consistent with the hypothesis that hRPE cells are competent to be a source of proteins found in drusen deposits. Our results suggest that donor-specific genetic and environmental factors influence the RPE stress response. ARPE-19 cells appear to be less representative of AMD-like changes than RPESC-derived hRPE. This adult stem cell-based system using chronic TBHP treatment of hRPE represents a novel *in vitro* model useful for the study of drusen formation and dry AMD pathophysiology.

## INTRODUCTION

AMD is a highly prevalent disease that is the leading cause of vision loss for patients over 65 years of age in first world countries [1-6]. An early sign of AMD is the formation of sub-RPE deposits termed drusen, which can accumulate over time leading to RPE dysfunction, cell death and loss of central vision. The majority of AMD patients, approximately 85%, have the ‘dry’ form of the disease, which is associated with drusen and defined by the absence of neovascular ingrowth. A minority of patients, approximately 15%, have ‘wet’ AMD in which choroidal neovascularization invades through the Bruch's Membrane resulting in macular edema and rapid central vision loss [7].

Drusen are the hallmark contribution to the diagnosis of dry AMD, and significant progress has been made to describe their mechanism of production and molecular composition [8-12]. Proteomic analysis of human drusen has identified a number of proteins that differ in expression in patients with and without an AMD diagnosis [11-13]. Among the most common proteins identified in drusen from AMD donor eyes are: βB1-crystallin, Clusterin (APOJ), Complement Component 9 (CC9), αB-crystallin, βA3/βA4-crystallins, βB2-crystallin, βS-crystallin, Histone H2A2 (H2AE), Tissue inhibitor of metalloprotease 3 (TIMP3), Vitronectin, and Annexin 2 [11]. Drusen from donors without a diagnosis of AMD had lower to undetectable levels of crystallins and proteins related to amyloid-β (Aβ) production [11-14].

The cell type responsible for the formation of drusen components has been debated and remains unclear. Electron micrographs have shown that the RPE can exude cell components from their basal membranes on top of and into drusen, suggesting that drusen are, at least in part, the products of RPE cells [15,16]. In a prior study, we found that αB-crystallin was highly expressed in RPE cells immediately adjacent to drusen, further demonstrating that in AMD, the RPE cells change to express proteins associated with the disease [17]. Evidence for RPE production of drusen proteins during AMD progression is accumulating [12,18-21]. Cultured fetal human RPE exposed to human serum were recently found to upregulate apolipoprotein E (APOE), APOJ, Vitronectin, and serum amyloid P (SAP), with evidence of complement activation [19].

Chronic oxidative stress is understood to be an important contributing environmental factor to the development of AMD [22]. Single exposure of cultured primary hRPE cells to hydrogen peroxide for 24 hours caused an upregulation of inflammatory factors IL-6, NF-κB, and phosphorylation of p38 MAPK, ERK, and JNK [23]. Other studies have used ARPE-19 cells, a transformed cell line originating from the RPE of a 19-year-old adult male donor [24], to study the impact of oxidative stress. Several similarities and differences between hRPE and ARPE-19 have been documented previously [25-27]. ARPE-19 cells exposed to single or repeated (up to 5 days) oxidative stress treatment upregulate several anti-apoptotic, inflammatory and DNA-repair proteins, consistent with some of the pathological changes associated with AMD [23,28-33]. However, upregulation of the most common drusen-related proteins in AMD has not been reported in primary adult hRPE or in ARPE-19 cells in response to oxidative stress.

In this study, we used a recently described adult stem cell present within the human RPE layer to produce homogenous hRPE cultures [34]. These RPESCs, which constitute roughly 10% of primary human RPE isolates, can be activated to self-renew, expanding into a large cell population that can be differentiated into progeny exhibiting the characteristics of mature RPE (hRPE). The ability to generate highly pure RPESC-derived hRPE cultures improves the interpretation of results.

Confluent cultures of RPESC-derived hRPE cells were subjected to a multi-day exposure to TBHP, shown previously to activate senescence in ARPE-19 [28]. TBHP exposure has been demonstrated to disrupt junctional integrity of the RPE and cause lipid peroxidation of the membrane bilayer as well as the oxidation of glutathione, endoplasmic reticulum Ca^2+^ release, increased intracellular calcium ([Ca^2+^]_i_), and increased mitochondrial inner membrane permeability [35-42]. TBHP has also been demonstrated to oxidize lipids in the membrane bilayer, thereby inducing membrane leakage leading to apoptosis and cell lysis [43]. We hypothesized that repeated exposure to TBHP would model the chronic oxidative stress previously associated with AMD [22]. Here we compared, for the first time, single and multi-day exposure to TBHP in differentiated RPESC-derived hRPE from 6 donors and the ARPE-19 cell line to ascertain the effect on expression of some of the major proteins associated with drusen and AMD (Appendix A). This large group of proteins and transcripts identified from the literature is hereafter termed ‘drusen-related’ in this report.

Repeated stress treatment of hRPE *in vitro* was found to be more effective than a single treatment at inducing upregulation of the major AMD-associated drusen-related protein transcripts. In contrast, ARPE-19 cells under chronic stress conditions showed a different pattern of response, with many of the major drusen-related protein transcripts being down-regulated. The observation that highly pure populations of hRPE respond in this manner, even in the absence of choroidal endothelial cells or neural retina, supports the evidence that the RPE is an important source of drusen components.

## RESULTS

**Figure 1:**
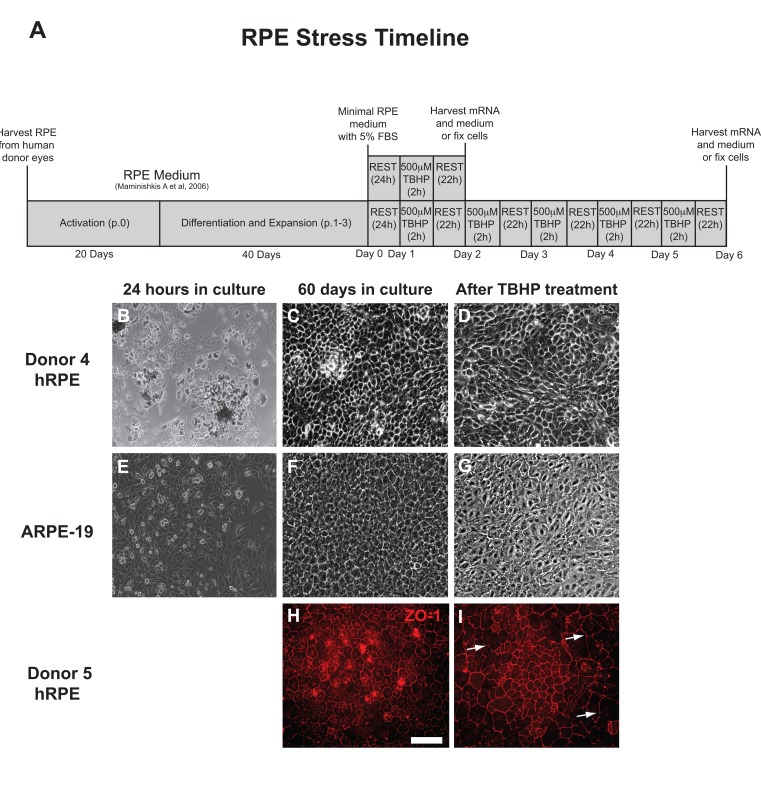
RPE cell morphology is disrupted by chronic TBHP treatment (**A**) Schematic of experimental design used for hRPE and ARPE-19 cells (p, passage; h, hours). hRPE from donors 4 (**B-D**) and 5 (**H,I**) as well as ARPE-19 (**E-G**) cells were collected, plated (**B,E**), and cultured for 60 days (**C,F,H**) prior to beginning an experimental course. TBHP treatment begins on day 1. ZO-1 (**H,I**), tight junction-associated marker (red). White arrows indicate regions suggestive of disrupted tight junctions. Scale bar, 100μm.

The experimental approach is presented as a diagram (Fig. 1A). hRPE cells were isolated and then plated into 24-well plates (Fig. 1B). The sub-population of RPESCs was activated to self-renew, dividing approximately once per day [34]. The resulting cultures were differentiated by lowering the serum concentration to produce a confluent culture of cells with the cobblestone epithelial morphology of mature hRPE (Fig. 1C) that exhibited a TER measurement of 200-250Ω ·cm^2^. The total time from initial donor harvest to the initiation of a stress experiment was 60 days (Fig. 1A). ARPE-19 cells were cultured in the same conditions as donor-derived hRPESCs and produced monolayers that closely resembled the cobblestone morphology of hRPE cells (Fig. 1E, F).

### Changes in cell monolayer integrity in response to oxidative stress

Light microscopic inspection revealed that hRPE and ARPE-19 cell morphology appeared minimally affected by one or two day exposure to TBHP, but after additional treatments, a moderate increase in cell size, loss of epithelial morphology, and disruption in ZO-1 immunoreactivity were observed, as illustrated at day 5 (Fig. 1D,G,I).

**Figure 2:**
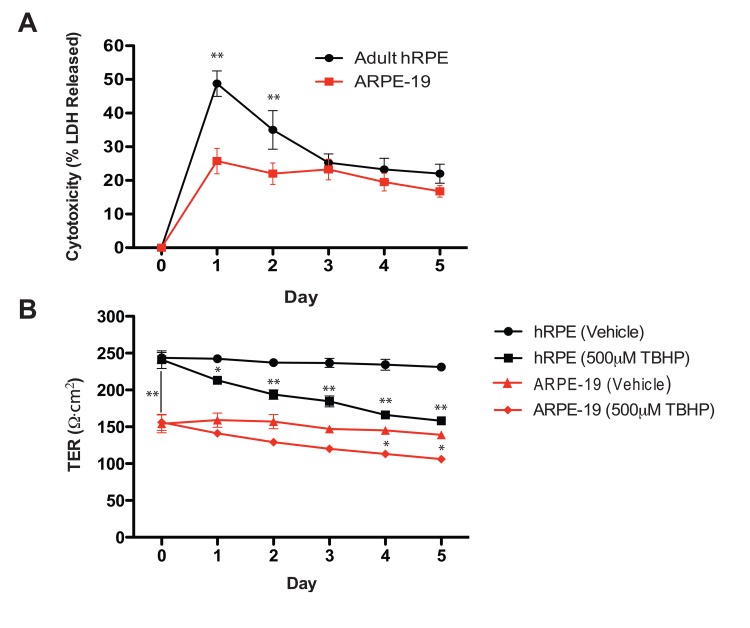
Oxidative stress reduces hRPE cell viability and disrupts transepithelial resistance (**A**) hRPE (n=4) release a greater percentage of total intracellular LDH in response to TBHP treatment than ARPE-19 cells (n=4) in the first two days, but both cell types responded similarly over the following days of TBHP treatment (Student's t-test, **, p<0.01). (**B**) The maximum achievable TER of the hRPE was significantly greater than that of the ARPE-19 under these culture conditions (Student's t-test, **, p<0.01). The TER decreased after multiple TBHP treatments (Student's t-test, *, p<0.05, **, p<0.01; Error bars represent SEM).

LDH release, which indicates plasma membrane compromise, was greatest following the first TBHP exposure for both hRPE and ARPE-19 cells (Fig. 2A). hRPE cells released significantly more LDH than ARPE-19 cells during the first two days (Student's t-test, **, p<0.01), but both cell types responded similarly over the next three days of TBHP treatment.

As a measure of integrity of the hRPE and ARPE-19 cell monolayers, we measured the TER daily, immediately prior to the next TBHP exposure. Substantial resistances (200-250Ω·cm^2^) such as those generated by the hRPE in this system are consistent with the generation of tight junctions [45], as is the observation of ZO-1 immunoreactivity (Fig.1H). Similar significant decreases in TER (Student's t-test, *, p<0.05, **, p<0.01) were observed in hRPE and ARPE-19 cells over the 5-day treatment period (Fig. 2B).

### Chronic TBHP treatment upregulates drusen-related protein and mRNA expression in hRPE cells

**Figure 3:**
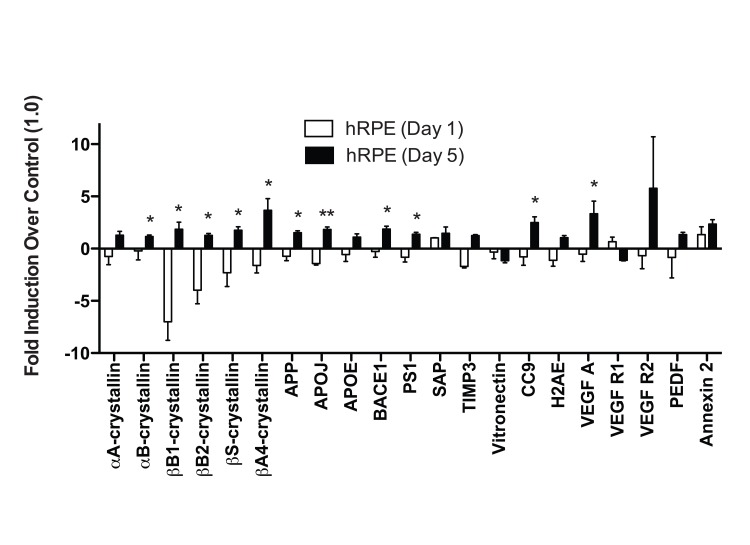
hRPE cells differ in response to acute and chronic oxidative stress hRPE cells were exposed to 500μM TBHP for 1 and 5 days. qPCR for drusen-related transcripts was controlled with S18 ribosomal RNA and GAPDH as housekeeping genes. None of the transcripts assessed were upregulated by hRPE after 1 day (n=4) of TBHP compared to controls. However, hRPE significantly upregulated 11 drusen-related transcripts following 5 days (n=6) of TBHP compared to hRPE treated for 1 day (Welsh's t-test, *, p<0.05; **, p<0.001). Error bars represent standard error of the mean (SEM).

The expression levels of 21 previously identified drusen-related protein transcripts (Appendix A) were measured after 1 and 5 days of 500μM TBHP exposure (Fig. 3). A single TBHP exposure was not sufficient to upregulate any drusen-related transcripts significantly when compared to vehicle-treated controls in the hRPE. However, following 5 days of TBHP treatments, 11 drusen-related transcripts were upregulated relative to hRPE treated with TBHP for 1 day (Welsh's t-test, *, p<0.05; **, p<0.01), including some that were unchanged or down-regulated after 1 day. The transcripts observed to be upregulated from day 1 to day 5 of TBHP in the hRPE were αB-, βB-1, βB2-, βS-, and βA4-crystallins, Amyloid precursor protein (APP), APOJ, β-secretase (BACE1), Presenilin 1 (PS1), CC9, and Vascular endothelial growth factor A (VEGF A).

To analyze total expression response profiles following 1 and 5 days of TBHP treatment across all six hRPE donors, we treated days of treatment as one variable and total change in protein expression (including transcripts that did not change significantly when compared individually) as a second variable, and performed a two-way ANOVA. This analysis revealed a highly significant difference in the expression level of this group of transcripts between 1 and 5 days of treatment (p<0.01).

**Figure 4:**
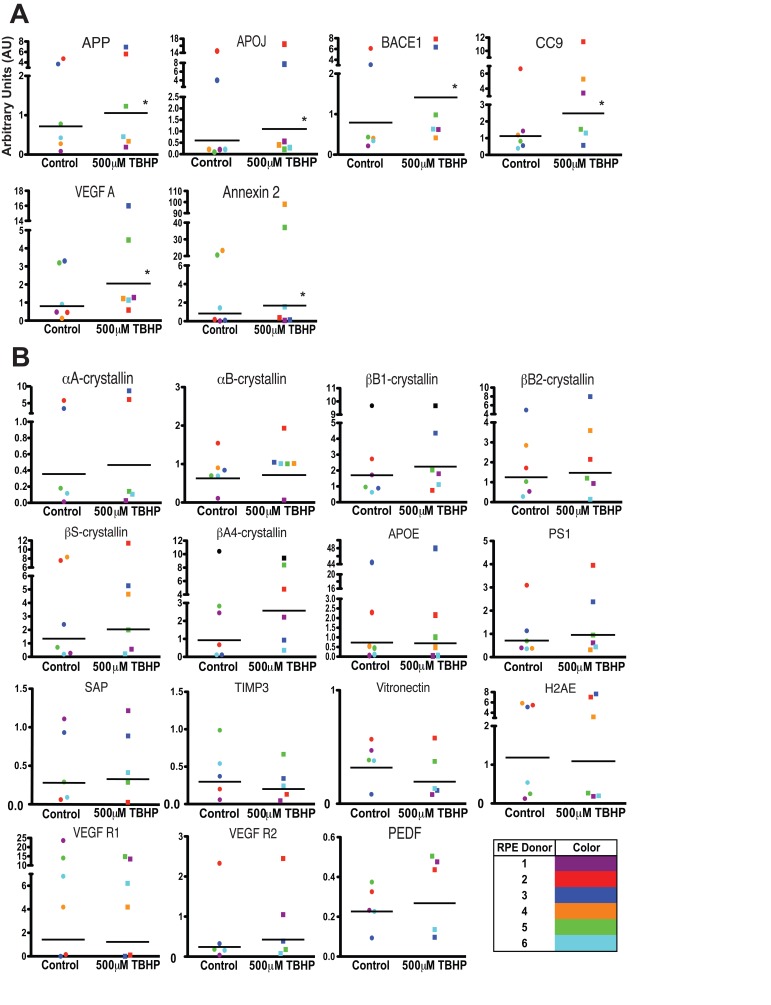
Individual donor variation in response to 5 days TBHP and a set of 6 consistently upregulated transcripts hRPE (n=6) were exposed to 500μM TBHP for 2 hours each day for 5 days and mRNA was quantified using qPCR. (**A**) Relative quantitation identified 6 transcripts with significantly higher mean expression levels after TBHP treatment compared to controls (Wilcoxon Matched Pairs Test *, p<0.05). hRPE mRNA was quantified relative to housekeeping genes (S18 ribosomal RNA and GAPDH) as well as to the mean of vehicle-treated control hRPE for each transcript across all donor cell lines. (**B**) 15 of the drusen-related transcripts did not show consistent upregulation after TBHP treatment. hRPE donors are indicated by color. The bar represents the geometric mean.

Interestingly, we found that there was considerable variability between the different donor hRPE expression responses (Table 2) to 5-day TBHP treatment (Fig. 4). Nevertheless, following 5 days of TBHP, the different donor hRPE cells consistently demonstrated a significant upregulation of 6 transcripts relative to vehicle-treated controls (Fig. 4A). While some of the hRPE upregulated more than these 6 (Table 2), only APP, APOJ, BACE1, CC9, VEGF A, and Annexin 2 were significantly upregulated across all 6 hRPE donors (Wilcoxon Matched Pairs Test, *, p<0.05). The remaining 15 transcripts showed a variable response depending on the individual donor (Fig. 4B).

**Table 1:** Demographic and Clinical Characteristics of RPE Cell Donors

Patient	Age	Race	Sex	Cause of Death	Eye Disease	Comorbidities	Medications
RPE 1	50	B	F	U	Cataracts	Type 2 Diabetes, Cirrhosis, Lung cancer, Colon cancer, Asthma, Schizophrenia, Sarcoidosis	Zyprexa, Prednisone, Lithium
RPE 2	85	C	F	MI	None	Vfib, Afib	Coumadin, Digoxin, Lasix
RPE 3	77	C	F	MI	None	Dementia, hypothyroid	Synthroid, Lipitor, Coreg, Metformin, Cozar, Zoloft, Coumadin
RPE 4	73	C	F	CHF	None	CVA, HTN, renal failure	U
RPE 5	76	C	F	Sepsis	None	Afib, Arthritis, AD, Hyperlipidemia, HTN, renal failure, PVD, CAD, Dementia, Asthma	L-dopa, Levophed, Ativan, Diltiazem, Fentanyl, Zocor, Effexor, Plavix, Metotprolol
RPE 6	63	C	M	EC	None	Pleural Effusion, Emphysema	Metoprolol, Celexa, Abilify, Oxycodone

Demographic and clinical characteristics of the primary adult hRPE cell donors used. (B,Black; C, Caucasian; M, Male; F, Female; U, Unknown; MI, Myocardial Infarction; CHF, Congestive Heart Failure; EC, Esophageal Cancer; RF, Respiratory Failure; HTN, Hypertension; Vfib, Ventricular Fibrillation; Afib, Atrial Fibrillation; CVA, Cerebrovascular Accident; AD, Alzheimer's Disease; PVD, Peripheral Vascular Disease; CAD, Coronary Artery Disease)

**Table 2:** Fold Drusen-related Protein Expression in Primary hRPE Cells Organized by Donor Relative to Control (1.0)

**Donor**	**αA-crystallin**	**αB-crystallin**	**βB1-crystallin**	**βB2-crystallin**	**βS-crystallin**	**βA4-crystallin**	**APP**	**APOJ**	**APOE**	**BACE1**	
hRPE 1	1.90	0.60	1.00	1.80	2.20	1.00	2.20	2.80	0.60	2.90	
hRPE 2	1.00	1.25	0.30	1.20	1.50	6.00	1.20	1.20	1.00	1.30	
hRPE 3	2.80	1.25	5.00	1.60	2.10	7.80	1.90	1.90	1.00	2.10	
hRPE 4	0.35	1.00	1.00	1.25	0.50	1.00	1.20	2.10	1.00	1.10	
hRPE 5	0.80	1.45	2.10	1.20	2.80	2.95	1.60	1.70	2.55	2.15	
hRPE 6	0.90	1.45	1.75	0.50	1.30	3.25	1.10	1.35	0.50	1.80	
ARPE-19	0.60	0.50	1.50	0.80	0.70	7.65	1.05	0.60	0.40	1.15	
**Donor**	**PS1**	**SAP**	**TIMP3**	**Vitronectin**	**CC9**	**H2AE**	**VEGF A**	**VEGF R1**	**VEGF R2**	**PEDF**	**Annexin 2**
hRPE 1	1.50	1.00	0.80	0.20	2.40	1.50	2.70	0.60	30.50	2.00	2.85
hRPE 2	1.30	0.30	1.00	1.00	1.60	1.30	1.35	0.90	1.00	1.50	2.20
hRPE 3	2.10	1.00	0.60	1.20	1.00	1.50	4.80	1.00	1.00	0.90	2.00
hRPE 4	0.85	1.00	1.00	1.50	4.70	0.50	8.70	1.00	1.00	1.70	4.10
hRPE 5	1.40	1.00	0.70	1.00	1.90	1.10	1.30	1.05	1.00	1.35	1.90
hRPE 6	1.20	4.50	0.45	0.35	3.30	0.35	1.20	0.90	0.50	0.60	1.10
ARPE-19	1.20	0.55	0.50	3.50	0.60	1.55	0.65	0.50	0.60	0.40	1.50

Fold change in drusen-related mRNA expression in hRPE cells organized by source following 5 days of TBHP treatment for 2 hours/day. Drusen-related transcript expression differs by individual. Gene expression is relative to the housekeeping genes S18 and GAPDH as well as to vehicle-treated control hRPE from the same donor.

### hRPE and ARPE-19 cells respond differently to oxidative stress

**Figure 5:**
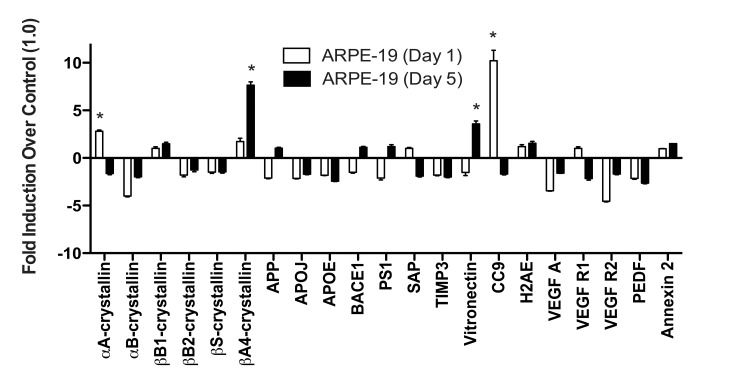
ARPE-19 cells do not upregulate most drusen-related transcripts after acute or chronic stress ARPE-19 cells were exposed to 500μM TBHP for 1 and 5 days. qPCR for drusen-related transcripts was controlled with S18 ribosomal RNA and GAPDH as housekeeping genes. ARPE-19 cells significantly upregulated αA-crystallin and CC9 after 1 day of TBHP treatment (Student's t-test, *, p<0.05). After 5 days of TBHP, ARPE-19 cells down-regulated αA-crystallin and CC9 to below control levels and significantly upregulated βA4-crystallin and Vitronectin (*, p<0.05). Error bars represent standard error of the mean (SEM).

When ARPE-19 cells were exposed to TBHP for 1 day, we detected only marginal differences in the drusen-related transcript expression profile (Fig. 5). αA-crystallin and CC9 were significantly upregulated compared to vehicle-treated controls 22 hours following this initial TBHP exposure (Student's t-test, *, p<0.05). However, both of these markers decreased to below control levels after 5 days of treatment (Fig. 5). Only βA4-crystallin and Vitronectin were significantly upregulated by the ARPE-19 compared to vehicle-treated controls after 5 days of TBHP treatment (Student's t-test, *, p<0.05). In contrast to the hRPE, the total expression response profile (Appendix A) of the ARPE-19 was not found to differ significantly between 1 and 5 days by two-way ANOVA (p=0.9161).

We then compared the mean expression level of each transcript in hRPE and ARPE-19 cells after 5 days of TBHP. Significant differences were observed in βS- and βA4-crystallins, APP, BACE1, VEGF A, PEDF, Annexin 2 (Welsh's t-test, p<0.05), αB-crystallin, APOJ, CC9 (p<0.01), and Vitronectin (p<0.001). We then compared the total drusen-related transcript expression profile (Appendix A) of each individual hRPE cell line to that of ARPE-19 cells after 5 days of TBHP and found that the hRPE expression response differed from that of the ARPE-19 in all cases (2-way ANOVA, p<0.01). This evidence demonstrated that none of the individual hRPE lines tested behaved similarly to ARPE-19 in this expression response assay.

**Figure 6:**
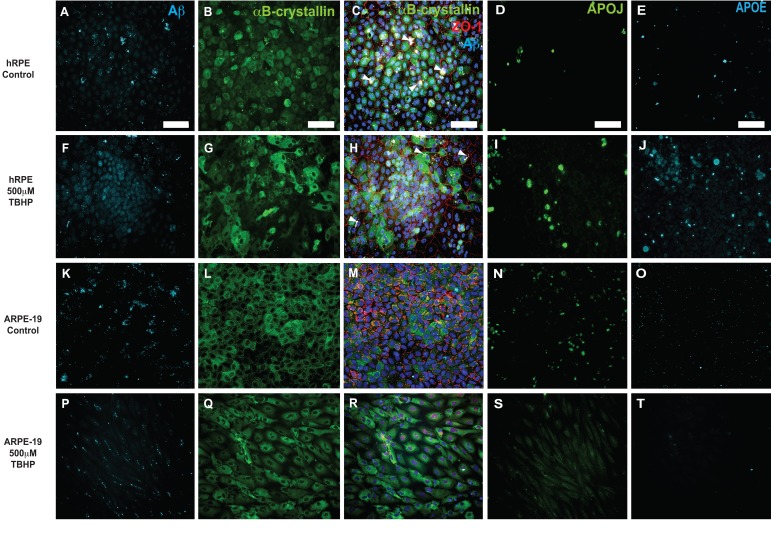
Immunocytochemical detection of drusen-related proteins expressed by hRPE and ARPE-19 cells Representative photomicrographs of vehicle-treated hRPE from donor 5(**A-E**) and ARPE-19 cells (**K-O**). hRPE (**F-J**) from donor 5 and ARPE-19 (**P-T**) cells were exposed to 500 μM TBHP or vehicle in medium for 2 hours/day for 5 days. Changes in cellular morphology can be seen from observing the changes in distribution of cytoplasmic αB-crystallin (**G,Q**) and the tight-junction associated marker ZO-1 (**H,R**). Increased expression of Aβ (**F**), APOJ (**I**) and APOE (**J**) was observed in hRPE exposed to TBHP, but not in ARPE-19. Expression is heterogeneous, with some cells expressing notably more than others in the field. Nuclei are labeled with DAPI (blue). White arrowheads indicate pigment deposits. Scale bar, 100μm.

### TBHP treatment results in altered drusen-related protein expression

To confirm that this treatment elicited changes in drusen-related protein expression, hRPE and ARPE-19 cultures were fixed following a 5-day TBHP exposure or vehicle and probed side by side for several of the drusen-related proteins (Fig. 6). hRPE and ARPE-19 cells exposed to 5 days of vehicle control typically had a consistent epithelial morphology with uniform ZO-1 immunoreactivity (Fig. 6C,M; ZO-1, red). αB-crystallin (Fig. 6B,L; green) was expressed in the cytoplasm in many untreated cells. Aβ (Fig. 6A,K), APOJ (Fig. 6D,N), and APOE (Fig. 6E,O) were expressed at relatively low levels in control conditions. In hRPE (Fig. 6F-J) and ARPE-19 (Fig. 6P-T) cultures exposed to TBHP for 5 days, the ZO-1 immuno-reactivity (red) was irregular (Fig. 6H) or absent (Fig. 6R), suggestive of disrupted tight junctions. Altered cellular morphology was further illustrated by the irregular distribution of αB-crystallin (Fig. 6G,Q), which appeared to be upregulated in some cells and not others. Expression of Aβ (Fig. 6F,P) and APOJ (Fig. 6I,S) was increased heterogeneously in the hRPE and decreased in the ARPE-19 cells. While the observed increase in Aβ expression in hRPE was primarily localized to the nucleus, extracellular and cytoplasmic deposits were also found (Fig. 6F). Expression of APOE (Fig. 6J,T) was not generally upregulated in this model, with the exception of hRPE from donor 5 (Fig. 6J). Visualization of APOE expression was minimal in the ARPE-19 cells (Fig 6O,T) under stress and control conditions. Hence, changes in the protein expression mirrored changes in transcript expression observed after TBHP exposure.

## DISCUSSION

This study demonstrates for the first time that hRPE cells respond to repeated TBHP exposure by changing to upregulate several proteins commonly found in AMD-associated drusen. These results establish the novel, RPESC-based hRPE *in vitro* system as a model of drusen formation and dry AMD with potential value as a tool for drug discovery.

Significant risk factors for the development of AMD include age, cigarette smoking, high body mass index (BMI) and consumption of high fat diets, all of which increase chronic stress to the body [52-57]. The RPE layer remains quiescent during life; the same cells present at birth are generally exposed to ≥50 years of oxidative damage from the environment before the onset of AMD. While it has long been suspected that the RPE cell layer expresses many of the proteins detected in drusen [12,15-19], the specific cell type or types responsible for the deposition of most of these proteins within drusen remains undetermined. Here we show that chronic exposure of hRPE cells to 500μM TBHP for 2 hours/day for 1-5 days induced cell dysfunction and damage that manifested in the upregulation of several drusen-related proteins, disrupted cell morphology, decreased cell viability and decreased epithelial integrity, all of which resemble the pathology associated with RPE dysfunction in AMD.

We assessed the TER and the level of LDH in the media comparing TBHP stress to vehicle-treated controls for hRPE cells. Interestingly, the large change in LDH release on day 1 (49% of the total available), which indicates severely compromised membrane integrity, appears at variance with the relatively small observed decrease in TER (12%). This apparent inconsistency could be explained if the mechanism of LDH release was temporary, for example via membrane blebs or transient pores present during TBHP exposure, followed by membrane re-sealing prior to the TER measurement. The precise mechanism of hRPE LDH release under TBHP conditions is not completely understood and is worthy of further investigation.

Upregulated expression of many drusen-related protein transcripts in stressed hRPE occurred in a time-dependent manner. Moreover, the pattern of expression was heterogeneous, with some cells expressing more protein than others. This observation is interesting in light of the heterogeneous distribution of drusen deposits observed in *vivo*. Similarly, in a prior study [17], we found αB-crystallin to be expressed heterogeneously in RPE cells surrounding drusen in AMD patients. The mechanisms behind these heterogeneous focal points of expression within the epithelial layer are unclear and remain an important point of future study.

The substantial variability in expression patterns observed between hRPE cell lines, presented in Table 2 and Figure 4, was surprising. The unique genetic makeup and history of exposure to environmental stress are likely contributors to the variable response of individual hRPE preparations *in vitro* [58], suggesting that studies of the RPE stress response should utilize several donors to account for donor variability. In addition, as our approach utilizes the sub-population of RPESCs as precursors for the hRPE cultures, some of the variation observed could be due to the particular sub-population of cells activated.

To further examine the variability in expression responses, we investigated whether the mean magnitude of drusen-related transcript expression change by the RPE was related to the age of the donors (Table 1). Age was not found to correlate with expression, but our study was limited by a sample size of six. Given a larger cohort of human donors, age, genetic, and environmental factors that have a significant impact on the expression of the transcripts might be identifiable using this model.

Despite the variability in individual hRPE response, we were able to identify a set of six drusen-related transcripts that were most likely to be upregulated by hRPE following a 5-day TBHP exposure across all individuals: APP, BACE1, APOJ, CC9, VEGF A, and Annexin 2. This set could be used to develop a reliable screen for AMD using donor-derived hRPE cells.

Of the six-transcript set, two, APP and BACE1, are involved in production of Aβ. Indeed, in this RPESC-derived hRPE disease model, we found consistent increases in Aβ peptide (1-40 and 1-42 isoforms) via immunocytochemistry, and in the mRNA transcripts of its precursor (APP) and processing enzyme (BACE1). Previous groups have demonstrated that Aβ is expressed in drusen [13,14,59,60], preferentially from patients with an AMD diagnosis [13], and that Aβ is cytotoxic to RPE cells and causes aberrant complement system activation [61-64]. While Aβ is traditionally thought of as a secreted or cytoplasmic product, we found a strong nuclear localization. This is consistent with prior studies showing that oxidative stress-induced DNA damage can lead to nuclear localization of intracellular Aβ42, binding to the p53 promoter, and apoptosis in guinea-pig primary neurons [65]. Hence, it is possible that there is a nuclear mechanism for Aβ-mediated RPE cell death and dysfunction.

The set also includes CC9, and while it is known that Aβ positively regulates the complement cascade [66,67] and CC9 expression [68] in other systems, this is the first time that the upregulation of CC9 by chronic TBHP exposure has been demonstrated, supporting the role of oxidative stress in modulating the complement system in hRPE. Annexin 2, a calcium-regulated membrane-binding protein, and APOJ (Clusterin) a molecular chaperone, were also among the set of 6 transcripts reliably upregulated by chronic TBHP in this study, although the mechanism responsible is unknown.

The sixth transcript of the set is VEGFA, known for its important pro-angiogenic role and contribution to the progression of AMD to the wet or neovascular form. While multiple pathways can lead to VEGF A upregulation in AMD, its upregulation in this model is possibly via TBHP-mediated upregulation of AP-1 [69-74] which is known to induce VEGF A [75]. Interestingly, both complement activation [76,77] and Annexin 2 activity [78] have been observed to increase VEGF A expression, suggesting that the set of 6 transcripts could positively interact to help produce a consistent upregulation of the set.

It was unexpected that αB-crystallin and TIMP3 were not consistently upregulated in hRPE cells (Table 2) as both of these proteins have been strongly associated with drusen [11,17]. Perhaps regulation of TIMP3 and αB-crystallin expression depends on signals other than oxidative stress, such as those from the choroidal vasculature or the retina.

Notably, our data show that the response of hRPE and ARPE-19 cells to the same oxidative stress regimen is significantly different, further reinforcing prior studies suggesting that the ARPE-19 cell line differs functionally from hRPE [25,26,79]. The ARPE-19 line was derived from the RPE of a 19-year-old male donor, raising the possibility that reduced lifetime environmental stress has resulted in a blunted stress response [24]. Other possibilities are that the mutations involved in the spontaneous immortalization of the ARPE-19 contribute to their resistance to oxidative stress, or that the ARPE-19 cell line may have come from a donor that was genetically less susceptible to oxidative damage. Our study suggests that ARPE-19 should be used cautiously for AMD disease modeling.

These findings are consistent with the hypothesis that cumulative oxidative stress is an important pathophysio-logic mechanism of drusen formation and AMD in the elderly. Further exploration and modulation of the stress response in this hRPE model may provide new targets for the development of novel therapeutics to slow the development of drusen as well as the advancement of dry AMD.

## METHODS

### RPE cell culture

RPE cells were obtained from 6 different adult cadaveric human eyes (Donor information summarized in Table 1) from NDRI, the Lions Eye Bank of Albany, or the New York Eye Bank under IRB-approved protocols, within 36-hours of the time of death. The anterior half of the eye was removed as were the vitreous and retina, thus isolating the posterior eyecup with the RPE/Bruch's membrane/choroid complex intact [34,44]. The eye cup was rinsed in sterile CMF-PBS and then incubated with dispase (1 mg/ml, Sigma-Aldrich) for 45-60 minutes at 37° C. The RPE tissue was collected in the dispase by denuding Bruch's membrane with a microsurgical angled, double beveled spoon blade, 3.0mm (Sharpoint). The resulting suspension of single cells and small sheets of RPE was pelleted by centrifuge (259gs for 5 minutes at 4° C). To separate single cells from RPE sheets, 2mL of 10% sucrose was added to the pellet and incubated for 15-20 minutes until the sheets fell to the bottom. The isolated sheets were then pelleted again by centrifugation (259g for 5 minutes at 4° C) and removed and resuspended in RPE medium [45] containing 10% fetal bovine serum (FBS), and plated at a density of approximately 150,000 cells/well on human placental extracellular matrix-(ECM, 10μg/ml, BD Biosciences) coated 24-well plates (Corning) (Fig. 1A). Once the primary (passage zero) cells reached confluence (20 days), the FBS concentration was reduced to 5%.

The expansion protocol involved dissociating primary cultures in 0.25% trypsin and plating onto ECM-coated 24-well plates (Corning) or 12-well polyester transwell membrane inserts (Costar, 0.4μm); thus the minor population of RPESCs was activated to proliferate and self-renew [34]. Once the cells reached confluence, the FBS concentration was reduced from 10% to 5% FBS to allow the RPESCs to differentiate into cobblestone hRPE (over 40 days), during which time the cells once again reached a confluent state and the transepithelial resistance (obtained weekly using a EVOM WPI Voltohmmeter) measured between 200-250Ω·cm^2^. The total time from initial collection of the RPE from donor eyes and the beginning of experimentation was approximately 60 days. 1 day prior to beginning stress experiments (Day 0), the medium was changed to minimal RPE medium consisting of DMEM/F12 (Corning), 2mM L-glutamine, 1:100 penicillin/streptomycin with 5% FBS. This medium was used for the remainder of the experiment. The hRPE used in all experiments were from passage 1-3, and not primary cultures (passage 0).

ARPE-19 cells were purchased from ATCC and cultured on placental ECM in the same manner as the donor-derived hRPE. ARPE-19 cells were used at passage 20-22 for experiments. The ARPE-19 cells were cultured until they reached a resistance of 150Ω·cm^2^ (TER measurement routinely achieved for ARPE-19 cells under these culture conditions).

### Chronic oxidative stress

For each experimental group, confluent hRPE cells in three wells of a 12-well transwell insert or a 24-well plate were fed with minimal RPE medium containing 500μM TBHP or vehicle for 2 hours per day for 1 and 5 days. Following each two-hour exposure, the RPE were washed twice with Hank's Buffered Salt Solution (HBSS, Gibco) and then rested in minimal RPE medium for 22 hours. This protocol for inducing chronic stress was adapted from Glotin et al., 2008 [28]. We utilized TBHP as an inducer of oxidative stress following prior studies of the RPE stress response [28,31].

### Transepithelial Resistance (TER)

Immediately prior to each treatment and at the end of the experiment, TER was measured in hRPE and ARPE-19 cells cultured in 12-well 0.4μm transwell inserts using the EVOM2 World Precision Instruments Voltohmmeter protocol. Resistance, measured in Ω·cm^2^, was recorded prior to the first TBHP treatment to confirm that all wells started at approximately the same TER, then 22 hours after each TBHP exposure to assess the changes due to oxidative stress. The TER of a cell-free ECM-coated transwell insert was found to be 30Ω·cm^2^, (not subtracted from presented TER values).

### Cell death analysis

Immediately prior to each TBHP treatment and following the measurement of TER, 300μL of medium from the culture wells was collected for quantification of lactate dehydrogenase (LDH) using the Roche Cytotoxicity Detection Kit Plus. LDH release experiments were controlled using medium from a vehicle-treated culture (0% cytotoxicity) and medium from a well of completely lysed cells (100% cytotoxicity) of the corresponding type. This method measures the level of LDH released into the media by porous or lysed cells and is used as a tool to assess cell membrane compromise that may or may not lead to cell death (Roche protocol).

### RNA isolation

Following the stress treatment and rest period, cells were incubated in RNA Protect (Qiagen) to attenuate endogenous RNAse activity and mRNA synthesis and scraped off the plate into a 1.5ml tube. Cells were centrifuged at 5,000 rpms for 10 minutes and the pellet was re-suspended in buffer RLT plus (Qiagen RNeasy Plus Micro/Mini Kits) with 2-mercaptoethanol (1:100 Sigma). RNA was harvested from the cells according to the protocol in the Qiagen RNeasy Micro/Mini Kits. Samples were passed through a gDNA eliminator column (Qiagen) to eliminate genomic DNA.

### Quantitative PCR (qPCR)

Purified total RNA was converted to cDNA using the high-capacity RNA to cDNA conversion kit (Applied Biosystems) followed by qPCR with gene-specific primers using a SYBR green reporter kit (Applied Biosystems). qPCR primers (Appendix A) were designed using the PrimerQuest online software from Integrated DNA Technologies and were designed to span exon-exon junctions within the coding region. Additional primers (Annexin 2, βB1-crystallin, PEDF, and BACE1) were obtained from the PrimerBank database, Harvard Medical School [46-48]. Expression was normalized to endogenous control genes ribosomal S18 [49] and GAPDH [50].

### Immunocytochemistry

Following the stress protocol, hRPE cells in 24-well culture plates were rinsed in calcium/magnesium-free phosphate-buffered saline (CMF-PBS) for 1 minute and fixed in 4% Phem-fix [51] for 20 minutes followed by two 20-minute washes in CMF-PBS. Cells were blocked and permeabilized with 5% normal goat serum (NGS) (Vector Laboratories) in PBS with 0.3% Triton (Sigma). Cells were incubated with primary antibodies in 5% NGS in PBS overnight at 4 degrees Celsius: αB-crystallin (Stressgen, 1:2500), Aβ4G8 (Millipore, 1:100), APOE (Millipore, 1:50), Clusterin 41D (Millipore, 1:100), ZO-1 (Invitrogen, 1:100). After rinsing again in PBS, cells were incubated with Alexa fluorescein-conjugated secondary antibodies (1:1200) (Invitrogen) with 5% NGS in PBS for 45 minutes at room temperature and shielded from light. After a further wash, cells were incubated with DAPI anti-nuclear probe (1 μg/μL; Sigma-Aldrich CO, St. Louis, MO) for 10 minutes in the dark at room temperature. After immunostained samples were cover-slipped with Vectashield mounting media (Vector Laboratories), two-dimensional phase and fluorescent images were acquired using an inverted microscope (Axiovert 200) with digital camera (AxioCam MRm) and AxioVision version 4.6.3 software (Carl Zeiss, Thornburg, NY). Immunodetection studies were controlled for background, autofluorescence, and fluorescence artifacts by omitting the primary antibody.

### Data Analysis

Graphpad Prism version 5.0b was used to generate graphs and analyze cytotoxicity, TER, and mRNA expression data. Error bars in all graphical figures represent the standard error of the mean (SEM). To visually display the different RPE conditions and sources on the same scale in Figures 3, the expression levels of each hRPE sample were first quantified relative to the mean delCT (the CT normalized to housekeeping genes) of the vehicle-treated control RPE from the same donor prior to comparing to other donors (Table 2). ARPE-19 cell expression data is graphically represented in Figure 5 in the same way.

To represent the variance in expression responses between the donors in Figure 4, the mean delCT values of TBHP or vehicle-treated control hRPE from each donor were quantified relative to the mean delCT of all untreated control hRPE from all donors for each mRNA transcript. Significance was determined using the nonparametric Wilcoxon Matched Pairs Test for comparing paired values of different numerical ranges, the Student's t-test, Welsh's t-test, and the two-way analysis of variance (ANOVA).

## Appendix A Quantitative PCR Primer Sequences

**Table:**

mRNA Transcript	Forward Primer (5'-3')	Reverse Primer (5'-3')	Source
αA-crystallin	GGTGTCTGTCTTCCTTTGCTTCCCTT	TAAGCTCTCCTGGCTGCTCTCT	NM_000394.2
αB-crystallin	AGGTGTTGGGAGATGTGATTGAGGTG	ACAGGGATGAAGTAATGGTGAGAGGG	NM_001885.1
βB1-crystallin	GTGCTCAAATCTGGCAGACC	GGAAGTTGGACTGCTCAAAGG	PBID: 21536279b1
βB2-crystallin	AAGGGAGAGCAGTTTGTGTTTGAG	CTTGTGCTCTTGGCTGTCCACTTT	NM_000496.2
βS-crystallin	GTATGAAACCACCGAAGATTGCCCTTCC	AGACACCCTCCAGCACCTTACA	NM_017541.2
βA4-crystallin	ATGGGATGGGAAGGCAATGAAGTAGG	CCGGAAATGTTTGTAGTCACCGGAATG	NM_001886.2
APP	CCGCTGCTTAGTTGGTGAGTTTGT	ACGGTGTGCCAGTGAAGATGAGTT	NM_000484.3
APOE	AACTGGCACTGGGTCGCTTT	GCCTTCAACTCCTTCATGGTCTCGT	NM_000041.2
APOJ	ATTTATGGAGACCGTGGCGGAGAAAG	CTGGTTACTTGGTGACGTGCAGAG	NM_001831.3
BACE	TCTGTCGGAGGGAGCATGAT	GTACAGCGAGTGGTCGATACC	PBID: 46255012b1
PS1	GAGTTACCTGCACCGTTGTCCTACTT	TGTGCTCCTGCCGTTCTCTATTGT	NM_000021.3
VEGF A	TCTTCAAGCCATCCTGTGTG	ATCCGCATAATCTGCATGGT	Kase S et al, 2010
VEGF R1	TCTGGGACAGTAGAAAGGGCTTCATC	ACTGGGCGTGGTGTGCTTATTT	NM_002019.4
VEGF R2	CCTCTGTGGGTTTGCCTAGTGTTTCT	CCCTTTGCTCACTGCCACTCTGATTATTG	NM_002253.2
PEDF	TTCAAAGTCCCCGTGAACAAG	GTCATAGCCGAAGTTGGAGAC	PBID: 54792142b1
CC9	ACGAACAGCAGGCTATGGGATCAA	CACGTTCCAAGGTCTTCGGTAGTATGT	NM_001737.3
Serum Amyloid P	AGACCTCAGTGGGAAGGTGTTTGT	CTTGGGTATTGTAGGAGAAGAGGCTGTAGG	NM_001639.3
TIMP3	TTCCCTTTGCCCTTCTCCTCCAATAC	CCTTGAGTCTATCTGCTTGCTGCCTTT	NM_000362.4
Vitronectin	TTTAGGCATCGCAACCGCAAAGG	GCCAGTCCATCCTGTAGTCATCATAGTT	NM_000638.3
Annexin 2	TGTGGATCGAGATGCCAAAAA	CCCATTCCTTTGCAGGCTTT	PBID: 51896028b1
H2AE	TGCTGTTAGGAAGCCACTATGTCTGG	ACACGGCCAACTGGAAACTGAA	NM_021052.2
Ribosomal S18	GATGGGCGGCGGAAAATAG	GCGTGGATTCTGCATAATGGT	Samovski D et al, 2009
GAPDH	ACAGTCGCCGCATCTTCTT	ACGACCAAATCCGTTGACTC	Kase S et al, 2010
